# Mother’s IPV, Child Maltreatment Type and the Presence of PTSD in Children and Adolescents

**DOI:** 10.3390/ijerph14091077

**Published:** 2017-09-17

**Authors:** Maravillas Castro, Mavi Alcántara-López, Antonia Martínez, Visitación Fernández, Julio Sánchez-Meca, Concepción López-Soler

**Affiliations:** 1 Faculty of Psychology, University of Murcia, Murcia 30100, Spain; maracastro@um.es (M.C.); mavialcantara@um.es (M.A.-L.); amperez@um.es (A.M.); visifernandez@um.es (V.F.); jsmeca@um.es (J.S.-M.); 2 Spanish Association for the Development of Mental Health in Childhood and Youth, “I Want to Grow”, Murcia 30001, Spain

**Keywords:** post-traumatic stress disorder, PTSD, child maltreatment, child witnessed maltreatment, mother maltreatment, intimate partner violence, CPSS, childhood trauma, child exposure to violence

## Abstract

This correlational cross-sectional study was designed to investigate whether the intimate partner violence (IPV) suffered by mothers (physical and psychological maltreatment), child eyewitness of psychological and physical maltreatment suffered by the mother, the neglect suffered by children, and the maltreatment (physical and psychological) directly suffered by children are statistically associated to post-traumatic stress disorder (PTSD) symptoms exhibited by the child. In addition, the prevalence of child PTSD was estimated, as well as the concordance between the PTSD symptoms assessed by the Child PTSD Symptom Scale (CPSS) and the Diagnostic and Statistical Manual of Mental Disorders, Fourth Edition, *Text Revision* (DSM-IV-TR) criteria for child PTSD. The sample consisted of 152 Spanish children aged 8 to 17 and their mothers, who were recruited from Centers of Specialized Assistance for Women Victims of IPV. PTSD prevalence was 20.4%. The results of a canonical correlation analysis showed that the two types of maltreatment with the largest contribution to the canonical variable were physical maltreatment directly suffered by the child, and child eyewitness of physical maltreatment suffered by the mother. The potential developmental pathway of PTSD when both children and mothers suffer severe maltreatment needs to be examined, and this will contribute to the choice of the most effective type of specialized intervention.

## 1. Introduction

The World Health Organization (WHO) [1] states that violence against women is a public health problem. One of the most common forms of violence is intimate partner violence (IPV), which includes *“*physical violence, sexual violence, stalking and psychological aggression (including coercive tactics) by a current or former intimate partner (i.e., spouse, boyfriend/girlfriend, dating partner, or ongoing sexual partner)” [2] (p.11). According to the WHO [1], almost 30% of women worldwide who have had an intimate relationship during their lives, have at some point suffered physical and/or sexual violence by their partner.

IPV towards women is also suffered by sons and daughters, and is now recognized as a form of child abuse [3,4,5]. Several research studies suggest that many children are exposed to the IPV directed at their mothers [6,7]. The latest macro-survey on gender violence in Spain [8] indicates that 63.6% of women who had suffered violence at the hands of their partners or ex-partners stated that their children witnessed or heard some of the episodes of conflict.

The early studies on the parental role of aggressors addressed only the perspective that the abused women had of the role of the abuser in the interaction with the children. Some investigations have appeared in the last decade that address the perspective of the abuser. For example, Rothman et al. [9] report that biological fathers, compared to stepfathers, were more aware of the potential negative impact of their behavior on their children’s mental health and of the various long-term after-effects. Salisbury et al. [10], however, report that although fathers were aware that their children were being exposed to the conflicts between them and their partners, few believed that they would be affected by them.

It is well documented that exposure to IPV can have adverse effects on the mental health of children and adolescents, and increase the risk of developing psychopathology [11,12,13]. Several studies have found a significant association between post-traumatic stress disorder (PTSD) diagnosis in children and exposure to IPV [14,15,16,17]. United Nations Children's Fund (UNICEF) [18] also indicates that the risk of suffering other types of additional maltreatment increases in children exposed to IPV. When evaluated worldwide, the co-occurrence rates of child abuse in children exposed to IPV show a significant variability range between 30–60% [19,20,21]. Although the type of abuse most strongly linked to exposure to IPV has been physical, more recent research suggests that neglect and psychological abuse may also occur [22]. When there is a coincidence of IPV with other types of abuse, the cumulative effect almost doubles the likelihood of children and adolescents’ being diagnosed with PTSD, compared with children who have only been exposed to IPV suffered by their mothers [23]. It is generally accepted that exposure to IPV and other types of abuse is less frequent in the general population than in high-risk and clinical examples [24].

The prevalence rates of PTSD in children exposed to IPV vary widely in the different studies, ranging from 13% to 60% [25,26,27,28]. Among the factors contributing to this variability, we focus here on the type of sample studied and the coincidence with other types of abuse. With respect to the type of sample studied, some research has focused on children exposed to IPV who were in foster homes with their mothers in emergency situations. For example, Mertin and Mohr [27] found a wide range of post-traumatic stress symptoms in 56 children aged 8 to 16. According to the self-report of the children, 29% were in the clinical range proposed by the Trauma Symptom Checklist for Children (TSCC) [29], and 69% in the Children‘s Impact of Event's Scale (IES-8) [30]. Only 20% of the children fulfilled the criteria for diagnosis of PTSD according to Diagnostic and Statistical Manual of Mental Disorders (DSM-IV) [31], presenting re-experiencing symptoms (R), 91%, avoidance symptoms (AV) 20%, and hyperarousal (HI) 89%. On the other hand, Jarvis et al. [32] in a sample from emergency shelters emphasized that PTSD symptoms in children aged 6 to 12, using the Child Post-Traumatic Stress Reaction Index (CPTS-RI) [33] were moderately severe (40%), severe (50%), or very severe (10%).

Other authors have studied children exposed to IPV whose mothers were receiving attention under social services and/or specific intervention programs and had been referred to mental health centers due to severe psychological disturbances. For example, Lamers-Wikelman et al. [21] revealed higher prevalence rates of post-traumatic stress symptoms in children when information was provided by mothers (57%; children aged 2 to 12 years old) through the TSCYCY (Trauma Symptom Checklist for Young Children) [34], than when the children reported it themselves (8%, 8–12 years) with the TSCC (Trauma Symptom Checklist for Children) [29], but in both cases, percentages were higher than those obtained in the general population. However, these data present certain comparative limitations due to the different tools used to assess PTSD, and to the different age groups of the children studied. The results of the evaluation on the mother’s perception of how violence affects their children are an ongoing debate. A high proportion of women tend to overstate the information, while in other cases, the opposite occurs. [35,36]. On the other hand, children’s perception of IPV varies with age, and the effects of exposure to IPV can manifest themselves in different ways according to the developmental stage of the child [11]. Telman et al. [37] reported that 21% of children exposed to IPV between 8 and 12 years old (*N* = 120) showed clinical levels of symptoms of post-traumatic stress, according to the information provided by their mothers (TSCYC [34]). In Spain, Olaya et al. [38] selected a sample of 520 children between 8 and 17 years old who attended mental health centers, of which 100 had been exposed to IPV in their family (in 95.5% of cases, the victim was the mother). They used Diagnostic interview for children and adolescents (DICA-IV) as an evaluation instrument [39]. Results indicate that the group of exposed children was more likely to present PTSD diagnosis according to DSM-IV-TR [40] than those not exposed (6.8% vs. 1.3%). In the same line, Graham-Bermann and colleagues [16] evaluated PTSD in 218 children aged 5 to 13 years, almost all of whom lived in the family home, who had been exposed to IPV suffered by their mothers. Through interviews, it was seen that 25% of the children met the criteria for PTSD according to DSM-IV [31] in relation to the violent incident to which they had been exposed in the last year. 76% met the symptom criteria for re-experiencing, 34.7% met the symptom criteria for avoidance, and 31.4% met the symptom criteria for hyperarousal.

Children exposed to IPV may often experience other adverse life events [37,41,42]. All of this contributes to variations in the in the rates of PTSD prevalence. One example is found in the study by Graham-Bermann et al. [43], which found that those who had suffered some other traumatic event (for example, being in a serious accident, assaulted by a family member or stranger, sexually abused, witnessing a fire or explosion, or had had a life-threatening illness) as well as exposure to IPV presented more symptoms in each PTSD (re-experiencing, avoidance and hyperarousal). Furthermore, the prevalence rate of diagnosis PTSD was higher (28%) compared with those who had only experienced IPV (8%). Moreover, when maltreatment or another type of exposure to violence is added to exposure to IPV, the negative impact on children's lives can be enhanced [44,45,46]. As described above, some authors have confirmed a greater likelihood of children presenting PTSD in whom IPV and other types of abuse co-occur. For example, Telman et al. [37] studied the relation between intimate partner violence (IPV), child abuse and neglect, other traumatic experiences, and children’s post-traumatic stress (PTS) symptoms in 120 children, and found that suffering combinations of these experiences explained 27% of the variance in post-traumatic stress symptoms in children exposed to IPV who were in mental health centers.

Finally, it should be noted that various studies have been conducted to evaluate the psychological impact on children exposed to IPV based on the different forms of maltreatment suffered by mothers, and additional types of abuse suffered by children in this context. Results show common aspects when dealing with multiple forms of violence, while each of these forms has distinctive aspects that can affect people psychologically and interfere negatively in their lives. For example, McDonald et al. [47], in a work on children living in foster homes with their mothers, found that direct abuse toward the child by the mother’s aggressor was positively associated with both internalizing and externalizing problems. Other research [48,49] has pointed out that the psychological abuse suffered by the mother significantly affects the development of psychopathology in children, in both internalizing and externalizing problems. Likewise, a recent study by López-Soler and colleagues [50] considered five types of abuse (physical and psychological maltreatment suffered by the mother, physical and emotional neglect, physical and psychological maltreatment suffered by the child) in a sample of 189 children aged between 6 and 17 years exposed to IPV, who had been referred for psychological treatment. The results confirm that both exposure to physical abuse to the mother and direct emotional abuse experienced by children had a greater association with internalizing and externalizing problems in children exposed to IPV receiving psychological attention.

However, to the best of our knowledge, there are no published studies that analyze the association between mother’s IPV, child maltreatment type, and the presence of PTSD in children and adolescents exposed to IPV. We have found only the investigation by Sullivan and colleagues [51] that studies the relation between different forms of child maltreatment and PTSD severity, in regards to the total number and type of each of their symptoms according to DSM-IV [31], in a sample of 89 adolescents who had been admitted to hospital for serious psychological problems. Hence, the aim of the present study was to analyze the relationship between the different types of maltreatment suffered by the mother and the children who have been in homes where IPV has taken place, in the development of the PTSD symptoms. In particular, our hypothesis was that the frequency of the IPV suffered by the mother (physical and psychological maltreatment), and the neglect and the maltreatment (physical and psychological) directly suffered by the child are statistically positively associated to an increase in the probability of the child’s suffering PTSD. In addition, we were interested in examining child PTSD prevalence and how different types of mother and child maltreatment are related to each other, as well as the interrelationships between the different PTSD symptoms in the children. The results will have clinical implications for the prevention and intervention of children exposed to IPV who have suffered different types of maltreatment.

## 2. Materials and Methods

### 2.1. Sample of Participants

The sample of children and their mothers was recruited from the Centers of Specialized Assistance for Women Victims of IPV (CAVI: Centro de Atención Especializada para Mujeres Víctimas de Violencia de Género) in the region of Murcia (Spain). The sample comprised 152 children (55.9% male; 44.1% female) and their mothers (*N* = 126). The children were between the ages of 8 and 17 years (mean = 11.41 years; SD = 2.84). The mean age of the mothers was 38.65 (SD = 5.83), in a range of 26 to 54 years. Spanish nationals made up 88.9% of the mothers; 6.3% had no education, 46.8% had primary education, 37.3% had secondary education, and 9.5% had university education. In most cases (97.4%), the aggressor was the biological father of the child. On enrolment for this study, most of the mothers (94.1%) did not live with the biological father of the child and 25.2% had a stable partner. More than half (57.9%) of the children lived with their mother only, 42.1% lived with their mother and other relatives, 8.4% lived with their mother and a stable partner, and 5.3% lived with their mother and the aggressor.

The inclusion criteria for this study were: (A) the child had to live at home with his/her mother and be aged between 8 and 17 years; (B) the mother had to be a victim of intimate partner violence (IPV) and be receiving psychological, social, and legal help at a specialized IPV center for IPV; (C) the child had internalizing and/or externalizing symptoms of moderate or severe gravity that interfered in his/her personal, family, social and/or school adjustment, and (D) the child had to have been exposed to the IPV suffered by his/her mother, but did not meet the social exclusion criteria for referral to child protection services. Children with psychotic disorders, mental deficiency, and who had suffered sexual abuse were excluded. Child victims of sexual abuse have been referred to another specialized attention service. It was considered that a child had been exposed to IPV when he/she fulfilled one of the following conditions: the child is told or overhears conversations about the IPV suffered by his/her mother, the child faces changes in his/her life as a consequence of the IPV, the child sees some of the immediate consequences of the IPV, the child hears and/or directly witnesses the IPV, the child is forced to participate in IPV scenes, and the child verbally or physically attempts to stop the IPV scene [14,52,53].

### 2.2. Instruments

The severity of post-traumatic symptomatology in the children was assessed with the Spanish version elaborated by Bustos et al. [54] of the Foa et al. [55] Child PTSD Symptom Scale (CPSS). The CPSS includes 17 items that parallel DSM-IV-TR re-experiencing (five items), avoidance (seven items), and hyperarousal (five items) PTSD symptoms. Children indicate how often they have experienced each symptom in the previous month using a four-point Likert-type scale (0 = not at all; 1 = once a week; 2 = two to four times a week; 3 = five or more times a week). A total PTSD score (range = 0–51) is calculated by summing all items, whereas re-experiencing (range = 0–15), avoidance (range = 0–21), and hyperarousal (range = 0–15) severity scores are calculated by summing relevant subscale items. The CPSS has proved good psychometric properties [54,55,56,57]. With the current sample, coefficients alpha were 0.75, 0.72, and 0.71 for re-experiencing, avoidance, and hyperarousal, respectively, and .88 for the total scale.

The intimate partner violence (IPV) suffered by the mother was assessed by the *Inventario de Evaluación del Maltrato a la Mujer por su Pareja*, APCM (*Intimate Partner Violence to Woman Inventory*). This inventory evaluates the maltreatment that a woman has received from her partner. It consists of 56 items, through which two types of abuse are evaluated: psychological maltreatment, comprising 37 items (range 0–148); and physical maltreatment, made up of 19 items (range 0–76). The higher the score in the APCM, the greater the frequency of abuse. The items have a five-point Likert response format (0 = Never, 1 = Sometimes, 2 = Often, 3 = Enough, 4 = Always). The mother re-took the APCM to assess the extent to which her children had been exposed to the maltreatment that she had received. Four inventory scores are therefore obtained: psychological and physical abuse suffered by the mother, and the child's account of the psychological and physical abuse suffered by their mother. The APCM has good validity and reliability properties. In the study by Matud et al. [58], a Cronbach alpha of .94 was found for both the physical and psychological abuse subscales. With the current sample, coefficients alpha were .95 and .93 for psychological and physical maltreatment suffered by the mother, and .98 and .93 for witnessed psychological and physical maltreatment, respectively.

The maltreatment directly suffered by the child was assessed with the *Inventario de Evaluación del Maltrato a la Infancia*, ICMI (*Childhood Maltreatment Assessment Inventory*). The ICMI is an evaluation instrument developed by our team and based on more than 15 years’ experience working with family violence and child abuse, as well as in the questionnaires of Matud et al. [58], Oliván-Gonzalvo [59], and Walker [60,61]. It aims to evaluate four types of direct abuse experienced by the child, and is applied to the mother. The ICMI consists of 60 items: 17 items for physical maltreatment (range = 0–51), 31 items for psychological maltreatment (range = 0–93), 10 fo neglect (range = 0–30) and two for sexual abuse (range = 0–6). The items have a Likert-type response format, with four categories (0 = never; 1 = once each two or three months; 2 = several times a month; 3 = several times a week). The higher the ICMI score, the more direct abuse suffered by the child. The ICMI has good validity, and the alpha coefficients obtained in the current sample for the four subscales were 0.75 (negligence), 0.93 (psychological maltreatment), 0.91 (physical maltreatment), 0.72 (sexual abuse), and 0.94 (total scale). One of the selection criteria of our sample was that the child was not suffering sexual abuse; as such, this subscale was not included in the statistical analyses. In fact, the mean for the two items of sexual abuse was very low (mean = 0.10; SD = 0.60).

Finally, an ad hoc questionnaire was prepared in order to obtain sociodemographic variables from the mother and the child. Age and gender were recorded from the child. Details of the mother’s age, nationality, marital status, level of studies, and labor status were recorded, as well as who she lived with at the time of enrolment, and the identity of the aggressor (the biological father of the child or other).

### 2.3. Procedure

The mothers and children who participated in this study were recruited from the CAVIs (Centre for Specialized Care for Women Victims of Intimate Partner Violence), which are centers catering for mothers with children who have emotional and behavioral problems. Children whose mothers attended the CAVI were referred by the psychologist to the psychological intervention service for assessment and treatment. This service is provided by the Association for the Development of Mental Health in Childhood and Youth, which is called “I Want to Grow”, under an agreement signed in 2009 with the Women’s Institute of the Region of Murcia, Decree No. 256/2009, July 31 (Official Bulletin of the Region of Murcia No. 178, August 4) (today, the Head Office for Women and Equal Opportunities).

The children taking part in this study were recruited from the 471 cases referred to the service between July 2009 and September 2014. Of these, 319 were excluded for not meeting study criteria. Therefore, 152 cases were included in this study, of which 100% participated.

At the first appointment at the service, a psychologist performed an assessment protocol to diagnose the child’s family situation and clinical indicators. Both mother and child were interviewed by a psychologist. The mother was also asked to complete questionnaires on the history of the family abuse suffered and the symptomatology she perceived in her child. Clinical and psychometric tests were also done for various psychological disorders, such as anxiety, depression, and post-traumatic stress symptomatology.

In all cases, informed consent and confidentiality standards were implemented and clearly explained to the child’s legal guardian.

### 2.4. Statistical Analysis

Percentages were calculated to estimate prevalence rates and concordance among PTSD symptoms and the DSM-IV-TR criteria for PTSD. With the purpose of examining the inter-relationships between the maltreatment suffered by the mother and the child, on the one hand, and PTSD symptomatology exhibited by the children, on the other, Pearson correlation coefficients were calculated among the seven types of maltreatment suffered by the mother/child and the three PTSD subscales assessed with the CPSS. In addition, a canonical correlation analysis was performed in order to explore whether the different types of maltreatment suffered by the mother and the child were statistically associated to the PTSD symptomatology suffered by the children. In the canonical correlation analysis, the set of predictor variables was composed of the scores obtained by the mother regarding her frequency of psychological and physical maltreatment, the negligence suffered by the child, the psychological and physical maltreatment directly suffered by the child, and the witnessed psychological and physical maltreatment of the child. The set of dependent variables was composed of scores obtained from the children regarding three PTSD symptoms (re-experiencing, avoidance, and hyperarousal). IBM SPSS (version 19.0, IBM Corp., Armon, NY, USA) was used for all of the statistical analyses.

## 3. Results

### 3.1. Estimating Child PTSD Prevalence

One of our aims was to estimate the prevalence of PTSD. The diagnosis of complete PTSD was studied according to the clinical criteria of the DSM-IV-TR diagnostic manual [40], which requires persistent symptomatology of at least one symptom of re-experiencing, three of avoidance, and two of hyperarousal, given that for criteria compliance a score of two or higher was needed on the scale (minimum two times a week).

The PTSD prevalence obtained in the sample of 152 children for re-experiencing, avoidance, and hyperarousal, and by applying the DSM-IV-TR criteria for PTSD, was 20.4% (95% CI: 14% and 26.8%). For the re-experiencing subscale of the CPSS, prevalence was 57.9% (95% CI: 50% and 65.7%). For the avoidance subscale, prevalence was 26.3% (95% CI: 19.3% and 33.3%), and for the hyperarousal subscale, it was 36.8% (95% CI: 29.1% and 44.5%) (see Table 1).

The concordance between the DSM-IV-TR criteria and each PTSD symptom subscale was 20.4%. Similar concordance percentages were found among the three PTSD symptom subscales: 24.3% between re-experiencing and avoidance, 32.9% between re-experiencing and hyperarousal, and 21.1% between avoidance and hyperarousal (see Table 2).

### 3.2. Canonical Correlation Analysis

In order to examine the interrelationships between PTSD symptomatology in children and child and mother maltreatment, a canonical correlation analysis was conducted. Regarding child PTSD symptoms, all correlations among the subscales were positive and statistically highly significant (*p* < 0.001). The greatest correlation was that found between the PTSD re-experiencing and PTSD hyperarousal subscales (*r* = 0.723). Although to a lesser degree, correlations between re-experiencing and avoidance (*r* = 0.618) and between avoidance and hyperarousal (*r* = 0.620) were also of a large magnitude (see Table 3).

The Pearson correlation coefficients between the seven types of maltreatment considered in this study (psychological and physical maltreatment suffered by the mother, child eyewitness memory of psychological and physical maltreatment suffered by the mother, negligence suffered by the child, and psychological and physical maltreatment directly suffered by the child) were positive and statistically significant (*p* < 0.01). The strongest correlations were found between psychological maltreatment suffered by the mother and child’s eyewitness memory of psychological maltreatment suffered by the mother (*r* = 0.645), and between psychological and physical maltreatment directly suffered by the child (*r* = 0.708) (see Table 4).

The Pearson correlations between the different types of maltreatment and the child PTSD symptoms were positive, with the exception of two, generally indicating a positive, statistically significant correlation between the direct link between maltreatment and the existence of PTSD symptoms in the children. Physical maltreatment directly suffered by the child was of the type most strongly correlated with the three PTSD symptom subscales, with correlations of *r* = 0.304 for PTSD re-experiencing, *r* = 0.224 for PTSD avoidance, and *r* = 0.317 for PTSD hyperarousal. Psychological maltreatment directly suffered by the child also exhibited statistically significant relationships with the three PTSD symptom subscales, with correlations of *r* = 0.219, 0.163, and 0.220 for re-experiencing, avoidance, and hyperarousal, respectively. In contrast, child negligence was not statistically related to PTSD symptoms (Table 5).

The canonical correlation analysis yielded three functions with squared canonical correlations (*R*^2^_c_) of 0.161, 0.038, and 0.009 for each successive function, taking the three children PTSD symptom subscales obtained from the Child PTSD Symptom Scale as the dependent variables, and the seven types of child/mother maltreatment as predictors. Collectively, the full model across all functions (Functions 1 to 3) was almost statistically significant using the Wilks’s λ = 0.799 criterion, χ^2^(21) = 32.583, *p* = 0.051. Since Wilks’s λ represents the variance unexplained by the model, 1 – λ yields the full model effect size in an *r*^2^ metric. Thus, for the set of three canonical functions, the *r*^2^ type effect size was *r*^2^ = 1 – λ = 1 – 0.799 = 0.201, indicating that the full model explained about 20.1% of the variance shared between the variable sets. Only the full model (Functions 1 to 3) approached statistical significance (see Table 6).

Given the *R*^2^_c_ effects for each function, only the first function was considered noteworthy in this study, with a 16.1% of shared variance between PTSD symptoms and child and mother maltreatment. Table 6 presents the standardized canonical coefficients, structure coefficients, and squared structure coefficients for Function 1. The Function 1 structure coefficients show that the three PTSD subscales exhibited a relevant relationship with the canonical variable, although re-experiencing and hyperarousal exhibited the highest percentages of shared variance (81.9% and 85%, respectively). To a lesser extent, avoidance also showed a relevant percentage of shared variance with the canonical variable (27.8%). Re-experiencing and hyperarousal also had standardized canonical coefficients (Coef. = −0.582 and −0.646, respectively). The negative sign of re-experiencing and hyperarousal indicated a positive relationship between them in the canonical variable.

Regarding the predictor variable set, four types of maltreatment exhibited relevant structure coefficients: the child’s eyewitness memory of psychological and physical maltreatment suffered by the mother, and the psychological and physical maltreatment directly suffered by the child, with 20.1%, 33.4%, 25%, and 67.4% of variance shared with the canonical function, respectively. With the exception of child negligence, the different types of maltreatment exhibited negative structure coefficients, which indicated a positive relationship with the canonical variable. Looking at the standardized canonical coefficients, one sees that the two types of maltreatment with the largest contribution to the canonical variable were physical maltreatment directly suffered by the child (Coef. = −0.721), and the child’s eyewitness memory of physical maltreatment suffered by the mother (Coef. = −0.486). This canonical variable can be called ‘direct and witnessed maltreatment suffered by the child’.

## 4. Discussion

The main aim of the present study was to examine the relationship between the different types of abuse suffered by the mothers and by their sons and daughters at the hands of their partner or ex-partner, and the development of PTSD symptoms in the children exposed to IPV. The results of the canonical correlation analysis show a positive relationship between the frequency of the abuse suffered by children exposed to IPV and the symptomatology of post-traumatic stress. The type of abuse that had the greatest influence was physical maltreatment of the child, and the child’s exposure to physical abuse towards the mother, and secondly, psychological maltreatment directed at the child and exposure to psychological abuse towards the mother.

Numerous empirical and meta-analytic studies [11,16,17] have found a strong link between exposure to IPV and PTSD symptoms. Our results also point to this association in children exposed to IPV who present psychological problems and have been referred for treatment.

The prevalence rates of PTSD obtained were 20.4%, similar to the percentage of PTSD found in the works of Mertin and Mohr [27], which was 20%, and Graham-Bermann and colleagues [16], 25%, and much higher than those found by Olaya et al. [38] in a clinical sample (7.8%). As for PTSD symptoms, the highest prevalence rate found was in re-experiencing (57.9%), followed by hyperarousal (36.8%) and avoidance (26.3%), coinciding with Mertin and Mohr [27], and similar to the data reported by Graham-Bermann et al. [16].

The correlations between the seven types of maltreatment studied (physical and psychological abuse suffered by the mother, exposure of the child to physical and psychological abuse suffered by the mother, neglect suffered by the child, and physical and psychological abuse suffered directly by the child) were positive and statistically significant (*p* < 0.01). These results were consistent with López-Soler and colleagues [50], which also found significant relationships between all types of abuse experienced by children exposed to IPV (physical and psychological mistreatment suffered by the mother, physical and emotional neglect suffered by the child, and physical and psychological abuse also suffered by the child). However, to date, no empirical evidence has been found in the published scientific literature to examine the association between the types of abuse directly suffered by children exposed to IPV and exposure to abuse of the mother.

The strongest correlations found in this work were between the psychological and physical abuse suffered directly by the child, and between the psychological abuse suffered by the mother and exposure to psychological abuse suffered by her.

Finally, calculating Pearson's correlation coefficients among all types of maltreatment studied and PTSD symptoms showed that all but two correlations (neglect of the child and psychological maltreatment of the mother) were positive, which indicated a direct relationship between abuse and the presence of PTSD in children. Physical abuse most strongly correlated with PTSD, followed by psychological abuse. Physical abuse presented a strong correlation with the three PTSD symptoms, and was stronger with hyperarousal, while psychological abuse was more related to avoidance. Child neglect did not present a positive relationship with PTSD.

As for the canonical correlation analysis carried out, the three PTSD symptoms were related to abuse, and especially, re-experiencing and hyperarousal. In relation to types of maltreatment, those that predicted the PTSD symptom in children were: the children’s account of physical and psychological maltreatment towards the mother, and the physical and psychological mistreatment suffered directly by the child.

It is difficult to compare the data obtained here since the only other similar study found [51] does not examine a sample of children exposed to IPV.

Some limitations of the results of our research need to be borne in mind. First, the sample included children whose mothers had been victims of IPV, and these children had been referred to a special clinical psychology service for evaluation and treatment because they presented behavioral/emotional problems. This circumstance limits the generalization of the findings to clinical sub-samples of children exposed to IPV with psychological problems, but not to all children exposed to IPV or to children who have been sexually abused, since in the sample the children had not suffered this type of abuse. Second, the interviews were conducted by mental health professionals only and information was only gathered on the maltreatment through their mothers, while the psychopathology of children was through self-report. Future research must incorporate protocol assessments from children about their own victimization into the evaluation, as well as clinical assessments of the psychologist. Third, other adverse experiences in the lives of children exposed to IPV and their mothers have not been taken into account. Last, our data are cross-sectional; hence, we are unable to make inferences about causal relationships between witnessing violence, various forms of violence experience, and mental health.

## 5. Conclusions

In summary, this study emphasizes the need to consider in depth the type of maltreatment that the child and mother have undergone in the context of IPV and its relation with the presence of PTSD in children and adolescents.

Our findings imply that children exposed to IPV and other types of child abuse who are in psychological treatment are more likely to suffer PTSD. Furthermore, studies on the effects of IPV often overlook the fact that exposure may be correlated with other types of abuse [22,43,62].

It is worth underlining the importance of these findings given the implications for referral for treatment. There is evidence that therapies that work with the mother and the child and those that work with children exposed to IPV are promising in terms of improved mental health and general wellbeing in the children [63,64]. It is important to note the need to focus on interventions with all family members when the relation with the aggressor does not suppose an important risk to the mother and child.

The findings of this work support the need to delve further into this field and examine the potential developmental pathway of PTSD when both children and mothers suffer severe maltreatment. Given the complexities involved, it would be necessary to combine the perspectives of trauma research and family research with the aim of advancing our understanding of PTSD symptoms in children during and following exposure to IPV. This line of research, despite being of prime interest, has not received much attention as yet.

## Figures and Tables

**Table 1:** Prevalence rates of child post-traumatic stress disorder (PTSD).

Prevalence Rates	Count (N = 152)	%	95% C.I.
Re-experiencing	88	57.90%	50.0–65.7
Avoidance	40	26.30%	19.3–33.3
Hyperarousal	56	36.80%	29.1–44.5
DSM-IV-TR criteria	31	20.40%	14.0–26.8

**Table 2:** Concordance among the different prevalence PTSD symptom subscales.

Prevalence Rates	1	2	3
1. Re-experiencing	100%		
2. Avoidance	24.30%	100%	
3. Hyperarousal	32.90%	21.10%	100%
DSM-IV-TR criteria	20.40%	20.40%	20.40%

**Table 3:** Pearson correlation matrix of PTSD symptoms in children measured with the Child PTSD Symptom Scale (CPSS) [55].

PTSD Subscale	1	2	3
1. PTSD-Re-experiencing	1		
2. PTSD-Avoidance	0.618	1	
3. PTSD-Hyperarousal	0.723	0.620	1
Mean	4.64	5.34	4.22
SD	3.47	4.10	3.45

All correlation coefficients were statistically significant (*p* < 0.001). *N* = 152 children. SD = Standard deviation.

**Table 4:** Pearson correlation matrix of child and mother maltreatment.

Type of maltreatment	1	2	3	4	5	6	7
1. Psychol. maltr.-mother	1						
2. Phys. maltr.-mother	0.37	1					
3. Witnessed psychol. maltr.	0.645	0.301	1				
4. Witnessed phys. maltr.	0.24	0.405	0.47	1			
5. Child negligence	0.278	0.247	0.278	0.304	1		
6. Child psychol. maltr.	0.528	0.401	0.581	0.219	0.452	1	
7. Child phys. maltr.	0.342	0.446	0.428	0.349	0.271	0.708	1
Mean	89.25	15.8	55.88	5.94	6.3	13.95	3.16
SD	32.8	15.21	39.7	9.91	5.59	15.07	5.94

All correlation coefficients were statistically significant (*p* < 0.01). *N* = 152 cases. SD = Standard deviation. Psychol. maltr.-mother: psychological maltreatment suffered by the mother. Phys. maltr.-mother: physical maltreatment suffered by the mother. Witnessed psychol. maltr.: child eyewitness of psychological maltreatment suffered by the mother. Witnessed phys. maltr.: child eyewitness of physical maltreatment suffered by the mother. Child psychol. maltr.: psychological maltreatment suffered by the child. Child phys. maltr.: physical maltreatment suffered by the child.

**Table 5:** Pearson correlation matrix of child PTSD symptoms and child–mother maltreatment.

Subscale	PTSD-Re-exper.	PTSD-Avoidance	PTSD-Hyperarousal
Psychol. maltr.-mother	0.152	0.125	0.138
Phys. maltr.-mother	0.185 *	0.082	0.131
Witnessed psychol. maltr.	0.165 *	0.129	0.176 *
Witnessed phys. maltr.	0.219 **	0.163 *	0.220 **
Child negligence	−0.024	0.107	−0.022
Child psychol. maltr.	0.206 *	0.210 **	0.201 *
Child phys. maltr.	0.304 ***	0.224 **	0.3170 ***

* *p* < 0.05. ** *p* < 0.01. *** *p* < 0.001.

**Table 6:** Canonical solution for types of maltreatment predicting child PTSD symptoms for Function 1.

Variable	Function 1		
	Coef	r_s_	$r_{s}^{2}$ (%)
Dependent variables:			
PTSD-Reexperiencing	−0.582	−0.905	81.9
PTSD-Avoidance	0.233	−0.527	27.8
PTSD-Hyperarousal	−0.646	−0.922	85
Predictor variables:			
Psychological maltreat.-mother	−0.181	−0.369	13.6
Physical maltreatment-mother	0.03	−0.432	18.7
Witnessed psychol. maltr.-mother	0.11	−0.448	20.1
Witnessed phys. maltr.-mother	−0.486	−0.578	33.4
Child negligence	0.534	0.131	1.7
Child psychological maltreat.	−0.105	−0.500	25
Child physical maltreatment	−0.721	−0.821	67.4

Coef = standardized canonical function coefficients. *r*_s_ = structure coefficients. Structure coefficients greater than |0.44| are underlined, as they represent a relevant contribution to the canonical variable. $r_{s}^{2}$ = squared structure coefficients.
